# Multiple genetic variants predict the progression-free survival of bevacizumab plus chemotherapy in advanced ovarian cancer: A retrospective study

**DOI:** 10.1097/MD.0000000000027130

**Published:** 2021-09-03

**Authors:** Jie Gao, Fang Li, Zihao Liu, Mengli Huang, Huoming Chen, Guoqing Liao, Jichang Meng, Qing Wang, Hui Zhao, Chenxi Li, Jing Ji, Shangli Cai, Nan Du

**Affiliations:** a Department of Oncology, First Affiliated Hospital of PLA General Hospital, China; b Department of Medical Oncology, Chinese PLA General Hospital, China; c The Medical Department, 3D Medicines Inc., Shanghai, China; d Department of Oncology, Chinese PLA Rocket General Hospital, China; e Department of Oncology, The 8th Medical Center of Chinese PLA General Hospital, China; f Department of Oncology, Bethune International Peace Hospital, Shijiazhuang, Hebei, China.

**Keywords:** bevacizumab, endothelial growth factor receptor, human epidermal growth factor receptor 2, next generation sequencing, ovarian cancer, predictive factor

## Abstract

Bevacizumab (BV) plus chemotherapy is broadly used in advanced ovarian cancer (OC). However, the efficacy of BV-based regimens for advanced OC patients is not satisfactory. Therefore, it is urgent to explore the predictive genetic biomarkers for BV.

Tumor tissues from advanced OC patients receiving BV-based regimens were analyzed with a 150-gene targeted panel for next generation sequencing. The associations between gene alterations or clinicopathology features and progression-free survival (PFS) were analyzed by Kaplan–Meier curves or Cox regression. The association of the genetic alteration in potential predictive genes and expressions of 11 vascular endothelial growth factor-related genes were analyzed in The Cancer Genome Atlas cohort using 292 OC cases.

Sixty two Chinese advanced OC patients treated with BV-based therapy were included. The median PFS of was 6.9 months, and objective response rate was 14.5%. In multivariate Cox regression analysis, the status of endothelial growth factor receptor (EGFR) (hazard ratio = 6.39, 95% confidence interval [CI] 2.25–18.13, *P* < .001) and human epidermal growth factor receptor 2 (HER2) (hazard ratio = 3.58, 95% CI 1.27–10.08, *P* = .016) were significantly correlated with PFS. MYC Proto-Oncogene amplification seemed to have a positive trend (hazard ratio = 0.21, 95% CI 0.05–1.02, *P* = .052). Moreover, EGFR and HER2 alterations were not prognostic factors of overall survival for OC in The Cancer Genome Atlas OC cohort. The vascular endothelial growth factor-related signature analysis indicated vascular endothelial factor A expression was upregulated with EGFR alterations (*P* = .034) which may be involved in BV resistance, and HER2 alterations were associated with hypoxia inducible factor 1 subunit alpha overexpression significantly (*P* = .029).

EGFR or HER2 alterations are negative predictors of PFS for OC patient treated with BV plus chemotherapy. Therefore, the clinicians may consider to use alternative regimens such as anti-EGFR or anti-HER2 targeted therapy instead of BV-based regimens on these patients when standard care fail.

## 1 Introduction

Ovarian cancer (OC) is one of the most common diagnosed cancer types among women all over the world^[1]^ and has the highest mortality rate among all gynecological cancers. Particularly, the annual mortality rate of OC in China has increased rapidly to 21.6% in 2015.^[2]^ Although early stage OC usually has a good prognosis, there is no effective method to detect early OC until now. As a result, most of the OC patients are found at advanced stage, which is associated with poor prognosis.

Recently, several novel targeted agents including bevacizumab (BV) have significantly improved the outcome of advanced OC.^[3]^ Bevacizumab is an angiogenesis inhibitor targeting vascular endothelial growth factor (VEGF), which results in the regression of tumor vasculature and the reduced formation of new blood vessels, leading to the suppression of tumor growth.^[3]^ BV combined with chemotherapy has been proved to significantly improve the progression-free survival (PFS) of OC patients compared with chemotherapy alone as shown by several randomized controlled trials.^[4–7]^ Therefore, the combination of BV and chemotherapy are now widely used in clinic. However, BV plus chemotherapy can not improve overall survival (OS) of OC patients.^[4–7]^

Unfortunately, 21.5% to 33% of OC patients failed to respond to BV plus chemotherapy as first-line therapy, and in the second line the proportion increased to 71.7%.^[4–7]^ Moreover, PFS varies highly among advanced OC patients, and there is no predictable biomarkers to select the group of patients who benefit more from BV-based treatment according to clinical guidelines. Some studies have shown that the polymorphism of vascular endothelial factor A (VEGFA) or vascular endothelial growth factor receptor (VEGFR) can successfully predict the therapeutic effects of BV in other cancer types, but is not confirmed by further studies.^[8–11]^ Moreover, Kommoss et al^[12]^ analyzed the correlation between the clinical outcomes in The International Collaboration on Ovarian Neoplasms 7 trial and the molecular subtypes of OC by RNA sequencing, and found 2 molecular subtypes (proliferative and mesenchymal) are associated with longer PFS from BV-based regiments.^[12]^ However, performing RNA sequencing requires high-quality tumor samples, therefore it is difficult to be applied widely in clinic. Therefore, it is urgent to explore predictable biomarkers in BV plus chemotherapy-treated OC patients.

With the rapid development of precision medicine, the next generation sequencing (NGS) has shown great value in the identification of potential biomarkers at genomic level.^[13]^ Using NGS panel, Hsu et al^[14]^ discovered that protein tyrosine phosphatase receptor type T and protein tyrosine phosphatase receptor type D are negative predictors of clinical outcomes in 36 metastatic colorectal cancer patients with BV plus chemotherapy. Herein, we conducted a 150-gene targeted panel for advanced OC patients who have received BV-based treatment, trying to seek potential genes associated with BV efficacy. The potential underlying mechanisms were further explored using the data from The Cancer Genome Altas (TCGA).

## 2 Methods

### 2.1 Patients

In this retrospective study, advanced OC patients treated with BV plus chemotherapy at the First Affiliated Hospital of PLA General Hospital (Beijing, China), from May 2015 to March 2018 were included. This cohort is named as Chinese OC cohort in the following sections. Eligibility criteria of this study include: pathological diagnosis of inoperable or metastatic OC; BV-based regimens; no previous systematic anti-angiogenesis therapy; measurable tumor lesions to assess therapeutic responses. BV at a dose of 300 mg were infused on day 1 and cycles were repeated every 21 days. PFS was used as the primary endpoint of the Chinese OC cohort in this study, according to clinical trials of BV in advanced OC. Response Evaluation Criteria in Solid Tumors criteria 1.1 was used to evaluated the objective response rate.^[15]^ The study was approved by the ethics committee of the First Affiliated Hospital of PLA General Hospital.

### 2.2 Tumor sequencing and analysis

Formalin-fixed paraffin-embedded (FFPE) tumor samples were obtained through operation or biopsy before BV treatment. Whole exons of 150 selected genes including frequently rearranged genes in OC were included in a multigene panel. Table S1, Supplemental Digital Content lists selected genes. The sequencing was performed by 3DMed Inc., College of American Pathologists (CAP)-accredited and Clinical Laboratory Improvement Amendments (CLIA)-certified Laboratory. The protocol of DNA sequencing by NGS was described previously.^[16]^ In short, hematoxylin and eosin (HE) staining was used to confirm the histological diagnosis, and to further eliminate samples with a total volume <1 mm or tumor cell percentage <20%. Fifty to 200 ng paired tumor and peripheral blood samples were used to extract genomic DNA, followed by breaking into ∼200 bp fragments. When targeted 150 genes were captured and sequenced in NGS platform, the pathogenic or likely pathogenic variants were identified with base single nucleotide variants, indel, copy number variants, and DNA arrangement.

### 2.3 TCGA data analysis

The survival, genomic, and transcriptomic data of 292 OC patients were obtained from TCGA. All the 292 OC patient were treated with platinum-based chemotherapy. The genes which could predict the efficacy of BV-based regiment significantly in the Chinese cohort would be analyzed with the progression-free interval (PFI) and overall survival in TCGA cohort to explore its prognostic valve. The expression of 11 genes (HIF1A, HIF2A, HIF3A, VEGFA, VEGFB, VEGFC, VEGFD, FLT1, KDR, FLT3, FLT4) which are involved in HIF-VEGF-VEGFR pathway^[17]^ were compared between 2 genotypes of these potential genes associated with BV plus chemotherapy efficacy which were identified in Chinese OC cohort.

### 2.4 Statistical analysis

PFS, PFI, and overall survival (OS) events were presented with Kaplan–Meier curves and compared with Log-rank test by GraphPad Prism v.6.0 (GraphPad Inc., San Diego, CA). The correlations between PFS and genes of interest or clinicopathological features were analyzed with Cox regression using Log-rank test by SPSS v. 17.0.0 (SPSS Inc., Chicago, IL). The factors with *P*-value <.05 in univariate Cox regression analysis would be included in multivariate analysis. The comparison of VEGF-related genes between genetic alterations group and wild-type group was performed by Mann–Whitney *U* test. *P* value <.05 was considered as significant.

## 3 Results

### 3.1 Baseline and clinical outcomes of Chinese OC cohort

**Figure 1:**
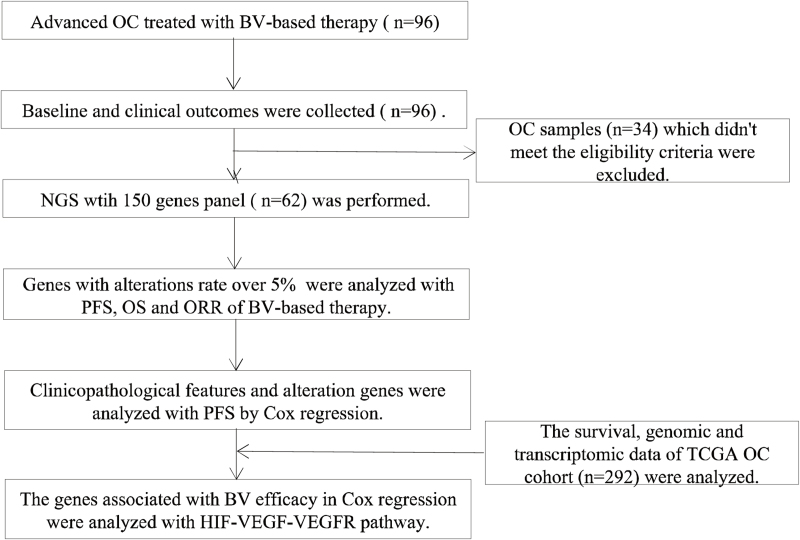
The study flow: patient inclusion, sample collections, and evaluable population.

Sixty two advanced OC patients were enrolled in the Chinese OC cohort, while 34 OC were excluded because they didn’t meet the eligibility criteria (Fig. 1). The clinical characteristics are summarized in Table 1. The median age of the patients was 59 years (ranging from 32 to 85 years) (Table 1). 93% of patients were histologically diagnosed as serous adenocarcinoma, of which 19.3% were diagnosed as stage III tumors and 80.7% were diagnosed as stage IV tumors (Table 1). The most common metastatic sites were peritoneal implantation (75.8%), followed by liver (38.7%) and lung (37.1%) (Table 1). In 69.4% cases, >3 metastasis sites were observed (Table 1).

**Table 1:** Baseline characteristics and clinical outcomes of advanced OC patients receiving BV-based treatment in Chinese OC cohort.

Variables	n	Percentage
Age, median (range), yrs	59 (32–85)	
Weight, median (range), kg	60 (49–77)	
ECOG		
1	55	88.70%
2	7	11.30%
Histology		
Serous adenocarcinoma	58	93.54%
Serous cystic adenocarcinoma	2	3.20%
Serous papillary adenocarcinoma	1	1.60%
Clear cell	1	1.60%
TNM stage		
IIIB	1	1.60%
IIIC	11	17.70%
IV	50	80.70%
Seroperitoneum		
Yes	7	11.30%
No	55	88.70%
Peritoneal implantation		
Yes	47	75.80%
No	15	24.20%
Liver metastasis		
Yes	24	38.70%
No	38	61.30%
Lung metastasis		
Yes	23	37.10%
No	39	62.90%
Bone metastasis		
Yes	4	6.50%
No	58	93.50%
Metastasis sites		
0	12	19.30%
2	3	4.80%
3	4	6.50%
>3	43	69.40%
Therapy lines		
1	1	1.60%
2	48	77.40%
3	12	19.40%
>3	1	1.60%
Chemotherapy plus bevacizumab		
Non-platinum	3	4.80%
Nedaplatin	47	75.80%
Carboplatin	7	11.30%
Lobaplatin	5	8.10%
Object response rate		
PR	9	14.5%
SD	51	82.3%
PD	2	3.2%
Progression-free survival, median (range), mo	6.9 (1.2–23.1)	
Overall survival, median (range), mo	NR (3.2–NR)	

ECOG = The Eastern Cooperative Oncology Group, NR = not reported, PD = progressive disease, PR = partial response, SD = stable disease, TNM = The Tumor, Node, Metastasis staging system.

77.4% of the patients received BV-based regimens as second-line therapy, while 19.4% had taken as third-line. The chemotherapy combined with BV was mostly platinum (95.2%), including nedaplatin (75.8%), carboplatin (11.3%), and lobaplatin (8.1%) (Table 1). According to Response Evaluation Criteria in Solid Tumors 1.1, 82.3% of patients were evaluated as stable disease (SD), among which 14.5% were partial response (PR) and 3.2% were progressive disease (PD). There were 43 PFS events with the median PFS period of 6.9 months (ranging from 1.2 to 23.1 months). There were 15 OS events (Table 1).

### 3.2 Correlation between gene alterations and PFS and ORR in Chinese OC cohort

**Figure 2:**
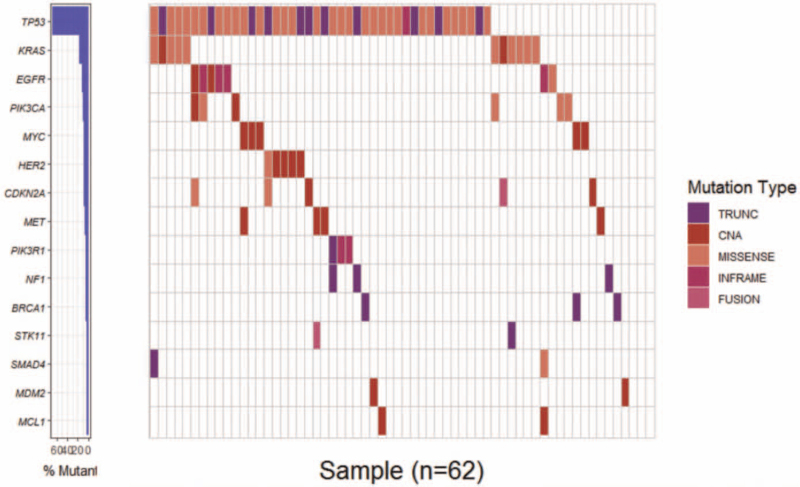
Molecular landscape of Chinese OC. The genetic alteration of tested genes in 62 advanced Chinese OC patients by targeted deep sequencing with 150-gene panel. Genes are ranked by frequency. Only genes with >3.2% frequency are shown. OC = ovarian cancer.

A total of 143 alterations, including 104 SNV and 37 copy number variation (CNV) were detected in Chinese OC cohort. The most frequent alterative genes were TP53 (69%), KRAS (17%), EGFR (11%), and PIK3CA (10%) (Fig. 2).

**Figure 3:**
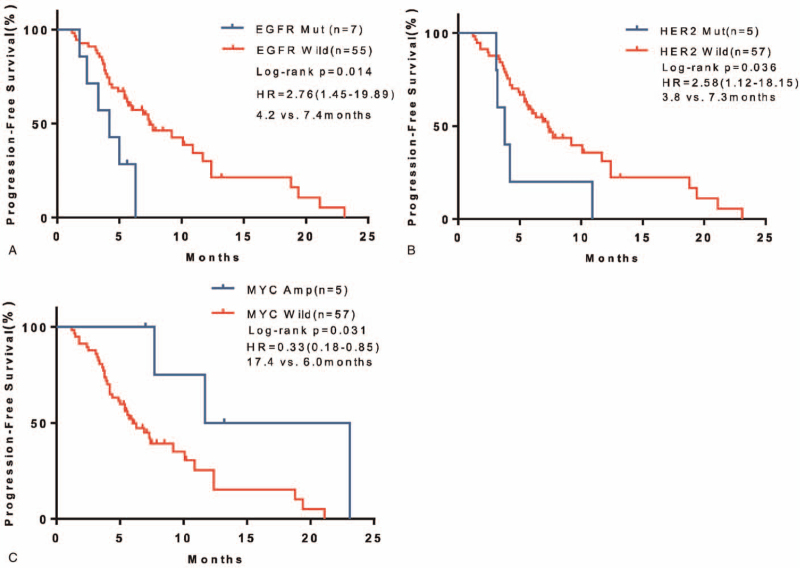
Association between gene alterations and PFS in Chinese OC cohort. (A–C) Kaplan–Meier survival curves of PFS. Comparisons of EGFR (A), HER2 (B), and MYC (C) alterations to corresponding wildtype were performed by log-rank test. EGFR = endothelial growth factor receptor, HER2 = human epidermal growth factor receptor 2, OC = ovarian cancer, MYC = MYC Proto-Oncogene, PFS = progression-free survival.

Eight genes (TP53, KRAS, EGFR, PIK3CA, HER2, MET, MYC Proto-Oncogene [MYC], CDKN2A) with over 5% alterative rate in 62 case Chinese OC cohort were analyzed when comparing PFS with Kaplan–Meier curves. Seven patients with EGFR alterations (2 amplification, 4 deletion, and 1 mutation) had significantly poorer PFS compared with EGFR wild-type patients (4.2 months vs 7.4 months, hazard ratio 2.76, 95% CI 1.45–19.89, *P* = .019) (Fig. 3A). What's more, 5 patients harboring another EGFR family member, HER2 (4 amplification and 1 mutation), also had significantly poorer PFS compared with wild-type patients (3.8 months vs 7.3 months, hazard ratio 2.58, 95% CI 1.12–18.15, *P* = .045) (Fig. 3B). Unexpectedly, 5 MYC-amplified patients showed improved PFS than MYC wild-type patients (17.4 months vs 6.0 months, hazard ratio 0.33, 95% CI 0.18–0.85, *P* = .049) (Fig. 3C). The other 5 genes which showed highest alteration rates, TP53, KRAS, PIK3CA, MET, CDKN2A were not significantly associated with PFS (Table S2, Supplemental Digital Content).

Moreover, there was no relationship between these genes with ORR (Table S2, Supplemental Digital Content). Except 1 patient with EGFR variant, all patients with EGFR and HER2 alterations failed to respond to BV-based treatment (Table S2, Supplemental Digital Content).

### 3.3 Cox regression analysis

In univariate Cox regression analysis, 13 clinicopathologic factors and 8 selected genes as described above were included. The results showed that 3 clinicopathologic factors (age, The Eastern Cooperative Oncology Group, and bone metastasis) and 3 genes (EGFR, HER2, MYC) have significant correlation with PFS (Table 2).

**Table 2:** Univariate and multivariate Cox regression analyses of potential factors affecting PFS in Chinese OC cohort.

	Univariate analysis			Multivariate analysis		
Parameter	HR	95% CI	*P*	HR	95% CI	*P*
Age	1.038	1.008–1.069	.013	1.031	0.995–1.069	.095
Weight	1.007	0.959–1.057	.787			
ECOG	3.357	1.448–7.784	.005	2.243	0.833–6.038	.11
Pathology	0.994	0.547–1.806	.983			
TNM stage	1.184	0.658–2.130	.573			
Seroperitoneum	2.333	0.961–5.661	.061			
Implanting metastasis of peritoneum	0.708	0.358–1.401	.322			
Liver metastasis	0.641	0.338–1.216	.174			
Lung metastasis	1.61	0.853–3.039	.142			
Bone metastasis	3.354	1.149–9.795	.027	4.215	1.351–13.148	.013
Metastasis sites	1.006	0.836–1.211	.946			
Prior system therapy lines	1.085	0.593–1.986	.791			
Chemotherapy(platinum based and non-platinum based)	0.655	0.379–1.130	.129			
TP53	1.554	0.725–3.331	.257			
EGFR	2.953	1.193–7.309	.019	6.392	2.254–18.128	<.001
KRAS	0.78	0.302–2.014	.608			
HER2	2.663	1.024–6.923	.045	3. 577	1.269–10.080	.016
PIK3CA	1.603	0.760–3.381	.215			
MET	1.775	0.627–5.029	.28			
MYC	0.235	0.056–0.991	.049	0.214	0.045–1.015	.052
CDKN2A	1.056	0.251–4.443	.94			

CI = confidence interval, ECOG = The Eastern Cooperative Oncology Group, HR = hazard ratio, OC = ovarian cancer, *P* = probability, TNM = The Tumor, Node, Metastasis staging system.

Based on univariate analysis results, the 3 genes (EGFR, HER2, MYC) and the 3 clinicopathological features (age, The Eastern Cooperative Oncology Group, and bone metastasis) were included in multivariate analysis. The results analysis showed that EGFR and HER2 were still significantly correlated with PFS (EGFR, hazard ratio 6.39, 95% CI 2.25–18.13, *P* < .001; HER2, hazard ratio 3.58, 95% CI 1.27–10.08, *P* = .016), and MYC amplification seemed to present a positive trend (hazard ratio 0.21, 95% CI 0.05–1.02, *P* = .052) (Table 2). Additionally, among the clinicopathological features, only bone metastasis was significant (hazard ratio 4.22, 95% CI 1.35–13.15, *P* = .013).

### 3.4 Association between EGFR and HER2 alteration and HIF-VEGF-VEGFR pathway in TCGA cohort

To explore the underlying mechanism, we further investigated whether EGFR and HER2 were prognostic factor in OC. For 292 OC in TCGA, both patients with EGFR or HER2 alteration had not significantly PFI or OS compared with EGFR or HER2 wild-type patients (Figure S1, Supplemental Digital Content), indicating that EGFR or HER2 alteration were not prognostic factor in OC, and the predictive value of EGFR and HER2 were associated with BV plus chemotherapy in OC.

VEGF is the target of BV, HIF-VEGF-VEGFR pathway plays a critical role in BV-based therapy.^[17]^ To test whether EGFR and HER2 are associated with HIF-VEGF-VEGFR pathway, we further checked the expression of HIF-VEGF-VEGFR related genes in TCGA cohort. EGFR mutation was found to associate with increased VEGFA expression (*P* = .034) (Fig. 4 A and B), while other 10 VEGF-related genes showed no relationship with EGFR genotypes (Fig. 4 A). VEGFA overexpression was previously reported to synergizes EGFR as one of the resistant mechanisms of BV therapy in other cancers,^[18]^ therefore it could explain why EGFR mutated OC had poorer PFS compared to EGFR wlidtype OC. At the same time, only the expression of hypoxia inducible factor 1 subunit alpha (HIF1A) was upregulated significantly in HER2-positive OC compared with HER2 wildtype OC (*P* = .029) (Fig. 4 C and D). These results suggested that EGFR and HER2 alterations were negative biomarkers for OC treated with BV plus chemotherapy due to that EGFR and HER2 alterations could active HIF-VEGF-VEGFR pathway and caused the resistance of BV.

**Figure 4:**
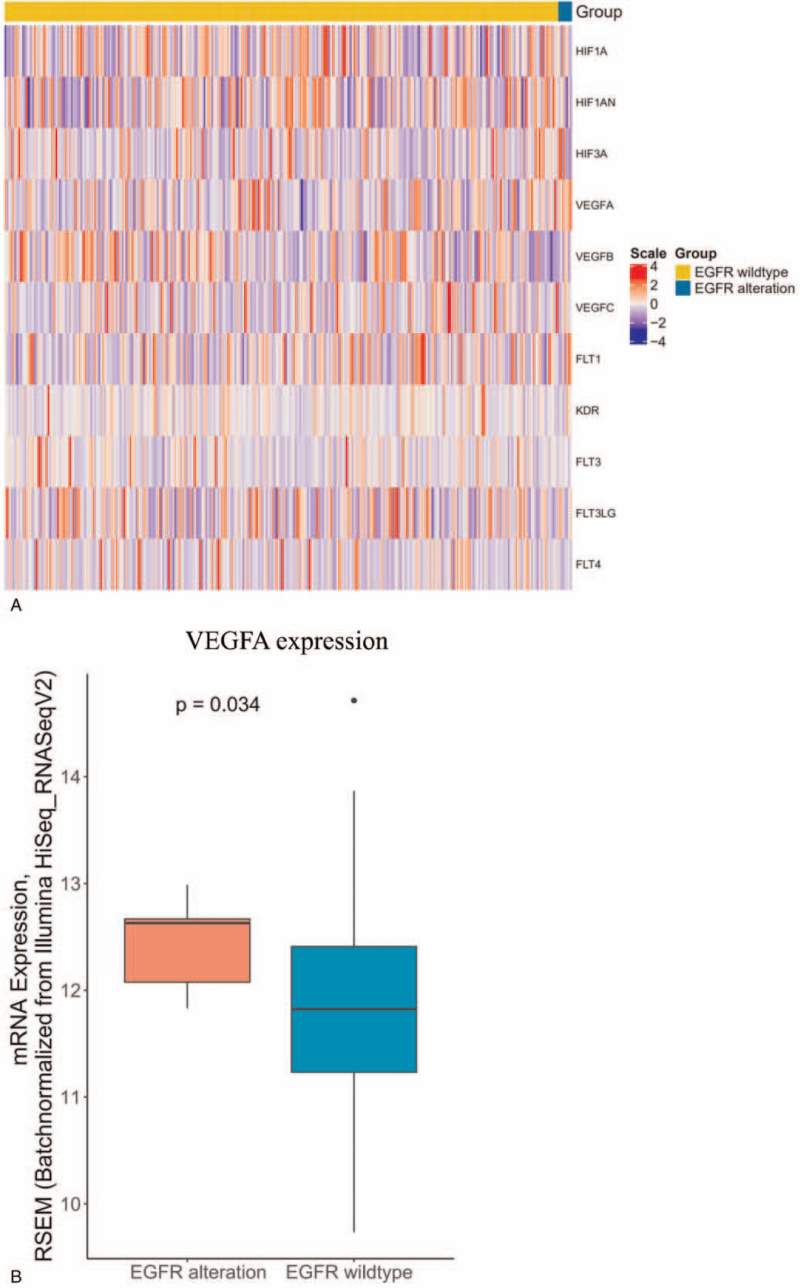
Association between gene alterations and HIF-VEGF-VEGFR pathway. (A) Heatmap of 11 VEGF-related genes expression between EGFR mutation and EGFR wild type in TCGA cohort. (B) Comparison of VEGFA mRNA expression between patients with EGFR mutation and EGFR wild type in TCGA cohort. (C) Heatmap of 11 VEGF-related genes expression between HER2 alterations and HER2 wild type in TCGA cohort. (D) Comparison of HIF1A mRNA expression between patients with HER2 alteration and HER2 wild type in TCGA cohort. EGFR = endothelial growth factor receptor, HER2 = human epidermal growth factor receptor 2, TCGA = The Cancer Genome Atlas, VEGF = vascular endothelial growth factor, VEGFR = vascular endothelial growth factor receptor.

**Figure 4 (Continued):**
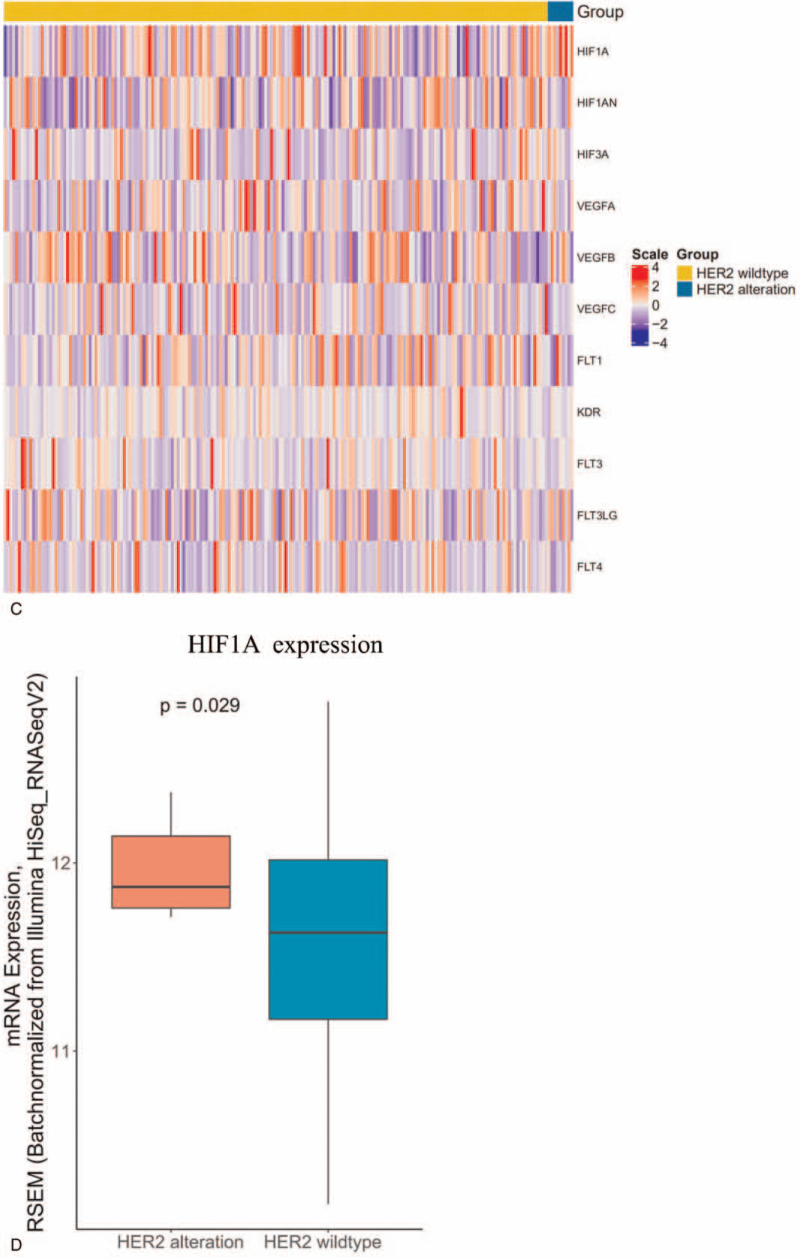
Association between gene alterations and HIF-VEGF-VEGFR pathway. (A) Heatmap of 11 VEGF-related genes expression between EGFR mutation and EGFR wild type in TCGA cohort. (B) Comparison of VEGFA mRNA expression between patients with EGFR mutation and EGFR wild type in TCGA cohort. (C) Heatmap of 11 VEGF-related genes expression between HER2 alterations and HER2 wild type in TCGA cohort. (D) Comparison of HIF1A mRNA expression between patients with HER2 alteration and HER2 wild type in TCGA cohort. EGFR = endothelial growth factor receptor, HER2 = human epidermal growth factor receptor 2, TCGA = The Cancer Genome Atlas, VEGF = vascular endothelial growth factor, VEGFR = vascular endothelial growth factor receptor.

## 4 Discussion

BV plus chemotherapy has been proved to improve PFS and ORR in OC compared with chemotherapy alone.^[4–7]^ However, some patients have rapid disease progression and cannot benefit from BV. Therefore, this treatment still belongs to “one size fits all” approach, and the clinically established predictive biomarker which can guide patient selection is still lacking. As far as we know, our study is the first one to identify the predict value of EGFR and HER2 genetic alterations in advanced OC patients receiving BV plus chemotherapy.

Our findings showed EGFR and HER2 were associated with PFS in OC patients treated with BV-based treatment in both univariate and multivariate Cox regression analysis. There are 2 types of possible underlying mechanisms how the alterations in these genes can affect clinical outcomes of BV plus chemotherapy: firstly, the alterations are associated with HIF-VEGF-VEGFR pathway and affect the efficacy of BV^[17]^; secondly, these genes are prognostic factor of OC and may have no relationship with BV. As a result, we analyzed the survival and VEGF-related signatures of OC with EGFR and HER2 using TCGA data. By analyzing PFI and OS in TCGA cohort, EGFR and HER2 alterations were both proved as not a prognostic factor in OC. Furthermore, EGFR and HER2 were found to be related with HIF-VEGF-VEGFR pathway. Activated EGFR was previously found to associate with VEGFA up-regulation in mouse model,^[19–21]^ which is one of the resistant mechanisms of BV. Therefore, Loizzi et al^[22]^ suggested that EGFR family members may be involved in angiogenesis and BV-based therapy in OC, but there has been no direct clinical evidence. In our study, we found patients with EGFR variants had shorter PFS in Chinese OC cohort, and further proved OC with activated EGFR were associated with higher expression VEGFA by analyzing TCGA data, suggesting that EGFR alteration is a negative predictor of BV plus chemotherapy through activating VEGF pathways in OC. At the same time, we found that expression of HIF1A was upregulated in HER2 activation OC compared with HER2 wildtype OC significantly. Larsen reported that the expression of HIF1A was significantly higher in BV-resistant colorectal cancer cell xenografts than BV-sensitive xenografts,^[17]^ indicating that upregulation of HIF1A was a potential resistant mechanism for BV. These results suggested that EGFR and HER2 alterations were negative biomarkers for OC treated with BV plus chemotherapy instead of negative prognostic factor for OC, and the underlying mechanism is that EGFR and HER2 alterations can active HIF-VEGF-VEGFR pathway and cause the resistance of BV.

Since OC patients with EGFR or HER2 variants benefit less from BV plus chemotherapy, these patients should be considered to use alternative regimens such as anti-EGFR or anti-HER2 target therapy. In our study, 3 of the 7 EGFR alteration cases are 19 exon deletion, which is recognized as a sensitive variant to EGFR tyrosinase inhibitor in lung cancer with 74% to 81% ORR^[23]^; Meanwhile, 4 of the 5 HER2 alteration cases are amplification, which can benefit from trastuzumab plus chemotherapy in breast, gastric, and other cancers.^[24–28]^ Therefore, anti-EGFR or anti-HER2 targeted therapy may become alternative treatments for those OC patients with activated EGFR or HER2 variants after the failure of standard regimens.

The major limitations of this study are the retrospective nature: small size. In this retrospective study, the median PFS and ORR of 62 advanced OC received BV-based regiment were 6.9 months and 14.3%, respectively. In 4 randomized controlled trials with BV-combined chemotherapy in OC, the range of median PFS was 6.7 to 19.8 months and ORR was 27.3% to 78.5%,^[4–7]^ which were better than our study. This discrepancy may be explained by: firstly, in 3 of the 4 randomized control trial (RCT) clinical trial, BV combined chemotherapy is the first-line treatment except AURELIA (PFS 6.7 months and ORR 27.3%), while 98.4% patients of our cohort received BV-based chemotherapy as second-line or third-line therapy; secondly, our study is a retrospective study thus the eligibility criteria was more relaxed than clinical trials such as the enrollment of aged patients (>65 years). Moreover, the combination regimens received by patients varied in drug, dose, and duration, which added to the heterogeneity of the study. Recent studies have shown that molecular markers show great value in diagnosing and prognostic malignant tumors.^[29–33]^ Therefore, a larger sample size study is worth being carried out to explore the preliminary conclusions of this study.

### 4.1 Conclusion

This is the first study to explore predictive biomarkers in advanced OC patients receiving BV-based therapy. We found that patients with EGFR or HER2 alterations had poorer PFS compared with wild-type patients. Therefore, the clinicians may consider to use alternative regimens such as anti-EGFR or anti-HER2 targeted therapy instead of BV-based regimens on these patients when standard care fail.

## Acknowledgments

Yuzi Zhang, Chan Gao, and Chuang Qi gave advices to this work.

## Author contributions

Nan Du and Shangli Cai designed and supervised this study. Jie Gao, Fang Li, Zihao Liu, Huoming Chen, Guoqing Liao, Jichang Meng, Hui Zhao, Chenxi Li collected clinical data. Mengli Huang, Jie Gao, Qing Wang, Jing Ji analyzed data.

**Conceptualization:** Shangli Cai, Nan Du.

**Data curation:** Jie Gao, Fang Li, Zihao Liu, Huoming Chen, Guoqing Liao, Jichang Meng, Hui Zhao, Chenxi Li.

**Formal analysis:** Jie Gao, Mengli Huang, Qing Wang, Jing Ji.

**Investigation:** Jie Gao, Fang Li, Zihao Liu, Huoming Chen, Guoqing Liao, Jichang Meng, Hui Zhao, Chenxi Li.

**Project administration:** Nan Du.

**Supervision:** Shangli Cai, Nan Du.

**Validation:** Jie Gao, Mengli Huang, Qing Wang, Jing Ji.

**Writing – original draft:** Jie Gao, Fang Li.

## Supplementary Material

Supplemental Digital Content

## Supplementary Material

Supplemental Digital Content

## Supplementary Material

Supplemental Digital Content
