# Supplementary material for: Multiple genetic variants predict the progression-free survival of bevacizumab plus chemotherapy in advanced ovarian cancer: A retrospective study
Source: Medicine (Baltimore). 2021 Sep 3;100(35):e27130. doi: 10.1097/MD.0000000000027130 (PMC8415939; doi:10.1097/MD.0000000000027130)
Supplement: Supplemental Digital Content [file medi-100-e27130-s003.doc]

**Supplementary Figure 1. Association between gene alterations and PFI and OS in TCGA OC cohort.** (A-B), Kaplan-Meier survival curves of PFI. Comparisons of EGFR (A), HER2 (B) alterations to corresponding wildtype were performed by log-rank test. (C-D), Kaplan-Meier survival curves of OS. Comparisons of EGFR (C), HER2 (D) alterations to corresponding wildtype were performed by log-rank test.
